# YOLO-BOS: An Emerging Approach for Vehicle Detection with a Novel BRSA Mechanism

**DOI:** 10.3390/s24248126

**Published:** 2024-12-19

**Authors:** Liang Zhao, Lulu Fu, Xin Jia, Beibei Cui, Xianchao Zhu, Junwei Jin

**Affiliations:** 1 College of Electrical Engineering, Henan University of Technology, Zhengzhou 450001, China; fll9681@163.com (L.F.); jx15090349719@163.com (X.J.); beibei.cui@haut.edu.cn (B.C.); 2 School of Artificial Intelligence and Big Data, Henan University of Technology, Zhengzhou 450001, China; xczhuiffs@163.com (X.Z.); jinjunwei24@163.com (J.J.)

**Keywords:** vehicle detection, YOLO-BOS, BRSA, ODConv, Shape-IOU

## Abstract

In intelligent transportation systems, accurate vehicle target recognition within road scenarios is crucial for achieving intelligent traffic management. Addressing the challenges posed by complex environments and severe vehicle occlusion in such scenarios, this paper proposes a novel vehicle-detection method, YOLO-BOS. First, to bolster the feature-extraction capabilities of the backbone network, we propose a novel Bi-level Routing Spatial Attention (BRSA) mechanism, which selectively filters features based on task requirements and adjusts the importance of spatial locations to more accurately enhance relevant features. Second, we incorporate Omni-directional Dynamic Convolution (ODConv) into the head network, which is capable of simultaneously learning complementary attention across the four dimensions of the kernel space, therefore facilitating the capture of multifaceted features from the input data. Lastly, we introduce Shape-IOU, a new loss function that significantly enhances the accuracy and robustness of detection results for vehicles of varying sizes. Experimental evaluations conducted on the UA-DETRAC dataset demonstrate that our model achieves improvements of 4.7 and 4.4 percentage points in mAP@0.5 and mAP@0.5:0.95, respectively, compared to the baseline model. Furthermore, comparative experiments on the SODA10M dataset corroborate the superiority of our method in terms of precision and accuracy.

## 1. Introduction

Currently, the acceleration of urbanization processes and the increase in vehicle ownership have exerted immense pressure on transportation systems, with traffic congestion emerging as a severe issue confronting cities. Prolonged periods of traffic congestion not only affect the efficiency of citizens’ travel and the quality of their lives but also exacerbate air pollution and energy consumption [1]. According to statistics released by TOMTOM in 2023, commuters in more than 10 global cities experience annual delays exceeding 100 h due to traffic congestion, with the problem expected to worsen. Consequently, the development of intelligent transportation systems (ITSs) [2] has become an urgent priority to alleviate traffic congestion and enhance transportation efficiency. Accurate identification of on-road vehicles, pedestrians, and other targets is a critical task within ITS and forms the foundation for achieving traffic intelligence.

However, in urban road traffic scenarios, the prevalence of occlusion and overlap phenomena significantly impacts the regression localization accuracy of vehicle detection and the classification results of various vehicle types, leading to frequent false detections and missed detections. To address these issues, this paper proposes YOLO-BOS, a novel vehicle-detection method based on a series of improvements to YOLOv8 [3], which effectively enhances the detection accuracy of small targets in complex road scenarios while maintaining a balance between accuracy and speed. The key improvements presented in this paper are as follows:

- To enhance the feature-extraction capabilities of the backbone network, we propose a novel Bi-level Routing Spatial Attention (BRSA) mechanism, which integrates the Bi-level Routing Attention (BRA) mechanism with the Spatial Attention Mechanism (SAM). This BRSA mechanism selectively filters features and dynamically adjusts the weights of spatial positions based on task requirements, facilitating focused attention on capturing intricate and subtle vehicle-related details and, therefore, enhancing the network’s ability to recognize salient features in complex backgrounds.
- In the head network, we integrate the Omni-directional Dynamic Convolution (ODConv), which dynamically learns complementary attention across four dimensions of the kernel space, enabling multi-dimensional information extraction. This enhances the convolutional neural network’s adaptability to variations in input data features, providing more flexible convolutional operations for handling complex and diverse data and thus better accommodating the intricate and changing conditions of road targets.
- To enhance the accuracy and robustness of vehicle target detection across various sizes, we introduce a novel loss function termed Shape-IOU. Building upon the traditional IOU, this loss function accounts for the impact of inherent bounding box attributes, namely shape and scale, on bounding box regression, therefore improving the localization precision of detected vehicles.

This article is structured into five sections. To understand the innovation of this research, Section 2 summarizes prior studies in the domain. Subsequently, Section 3 delineates the enhanced network architecture designed for more precise vehicle detection. In Section 4, the experimental setup and analysis are presented to evaluate the performance of the detection network. Finally, Section 5 consolidates the primary findings of the research and outlines directions for future investigation.

## 2. Related Work

### 2.1. Object Detection

Object detection is a critical task in computer vision aimed at identifying and locating objects of interest within images or videos. With the emergence of GPU technologies, deep learning-based object detection methods have progressively supplanted traditional approaches, becoming predominant. These methods are broadly categorized into two main types: two-stage and one-stage algorithms. Two-stage algorithms utilize a region proposal network (RPN) to propose candidate areas, followed by classification and regression processes. They typically feature complex network architectures and moderate detection speeds. Early two-stage detection algorithms employed selective search techniques, such as R-CNN [4], SPPNet [5], and Faster R-CNN [6]. Recent advancements in two-stage algorithms incorporate feature pyramid network (FPN) structures, which integrate spatial information from both high and low layers, as demonstrated in Mask R-CNN [7] and Libra R-CNN [8]. In contrast, one-stage detection algorithms employ simpler network architectures and achieve faster detection speeds, making them suitable for applications in scenarios like complex road scenes. Consequently, the pursuit of research on one-stage vehicle-detection algorithms holds paramount significance. The YOLO series of algorithms constitutes a paradigmatic example of one-stage detection networks, originally introduced by Joseph et al. in 2016 [9]. Following its inception, a multitude of research teams have embarked on investigations based on the YOLO framework, propelling its continual evolution and refinement. The sequential unveiling of YOLOv4 and YOLOv5 marked significant milestones in performance enhancement, solidifying the series’ prominent position within the realm of one-stage detection. Capitalizing on this robust foundation, subsequent iterations have undergone meticulous optimization. YOLOv8 refined the feature-extraction capabilities of YOLOv5 through the adoption of a decoupled head architecture and an anchor-free mechanism. YOLOv9, built upon the framework of YOLOv7, incorporated Programmable Gradient Information (PGI) alongside a Generalized Efficient Layer Aggregation Network (GELAN) structure. YOLOv10 innovatively eliminated the necessity for Non-Maximum Suppression (NMS) while introducing a sophisticated dual-label assignment strategy. As of the present, the YOLO series has advanced to its eleventh iteration, YOLO11.

### 2.2. Vehicle Detection

Currently, an extensive body of research has delved into developing algorithms for vehicle detection. Within the realm of two-stage detection frameworks, Zhang et al. [10] have augmented Faster R-CNN by leveraging inter-frame differences and spatiotemporal context, effectively mitigating the issue of missed detections in vehicle recognition tasks. Xu et al. [11] have further bolstered the accuracy of nighttime vehicle detection by integrating deformable convolution and soft-NMS within the Faster R-CNN architecture. Othmani et al. [12], on the other hand, have amalgamated the strengths of three distinct networks to enhance Faster R-CNN, yielding notable gains in both speed and accuracy. In the domain of one-stage vehicle target detection, Guo et al. [13] have significantly improved the accuracy of vehicle detection in remote sensing imagery by seamlessly incorporating an adaptive fusion module and a sub-pixel convolution module into YOLOv3. Zhang et al. [14] have addressed the challenge of detecting small targets by introducing bounding boxes with orientation angles, therefore enhancing detection precision. Additionally, Zhang et al. [15] have integrated a coordinate attention mechanism into YOLOv4, empowering the model to dynamically adjust weights and focus on salient regions within images. To optimize performance further, Wang et al. [16] and Chen et al. [17] have independently replaced the backbone network of YOLOv4 with CSPDarknet53-dcn and MobileNetv2, respectively, achieving notable improvements in both inference speed and detection accuracy. More recently, Miao et al. [18] presented the YOLO-VSF model by incorporating methodologies such as the VGG16 network and the SENet attention mechanism. The research endeavors surrounding YOLOv5 primarily focus on two pivotal directions: lightweight design [19,20,21] and enhanced small object detection [22,23,24]. Moreover, He et al. [25] have introduced the ShuffYOLOX model, a novel approach that leverages the ShuffDet and ECA modules within the YOLOX framework. This integration has yielded notable improvements. On the other hand, Gao et al. [26] have devised two innovative lightweight modules, DG and DS, which have achieved a remarkable 51% reduction in the number of YOLOX model parameters while maintaining its performance, showcasing the ongoing advancements in optimizing object detection models for efficiency and accuracy. Regarding YOLOv7, Wang et al. [27] have made seminal contributions by incorporating the Ghost module alongside improvements to the feature pyramid and loss function, culminating in the YOLOv7-GBW model. Concurrently, Zhang et al. [28] have reimagined the YOLOv7 backbone by reconstructing it with the Res3Unit structure and augmenting it with the ACmix module, significantly boosting the model’s precision in detecting minute objects within images. Similarly, Li et al. [29] presented the MST-YOLOv8 model through the integration of the C2f-MLCA structure and the ST-P2Neck structure. Meanwhile, Liu et al. [30] introduced a more streamlined vehicle-detection model by refining the detection head and upsampling architecture of YOLOv8. In recent years, object detection models leveraging the Transformer architecture, particularly Real-Time DETR (RT-DETR), have been widely adopted for vehicle detection owing to their superior real-time performance. Liu et al. [31] made further contributions by proposing the MDFD2-DETR model, which incorporates innovative feature de-redundancy modules and hybrid positional encoding strategies. Additionally, Jin et al. [32] developed a lightweight algorithm grounded in RT-DETR, specifically tailored to enhance the detection of distant vehicle logos.

## 3. Methods

### 3.1. Network Architecture

In complex scenarios of vehicle detection, including intersections and congested roads during peak traffic periods, existing detection models encounter substantial challenges. Due to mutual occlusion between vehicles, which results in incomplete visibility of vehicle bodies and consequently reduces their proportion in the image, feature information is more prone to being lost in shallow network layers. This often results in a heightened incidence of false positives and missed detections during target identification. Hence, it is crucial for detection models to bolster their capacity to extract feature information and augment the richness of valid data in order to enhance detection accuracy.

**Figure 1:**
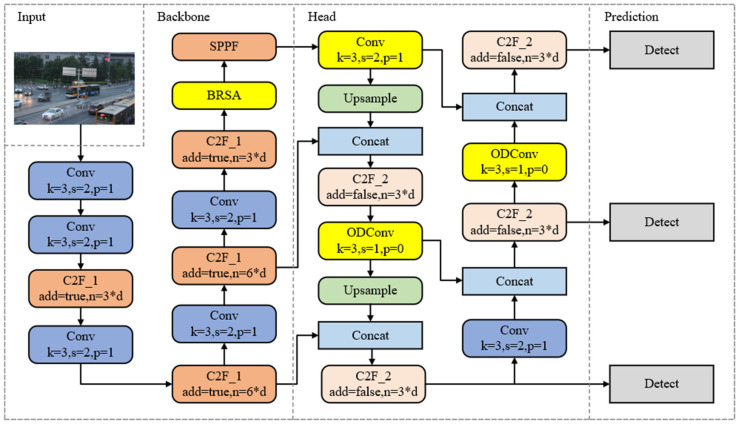
Structure diagram of the YOLO-BOS network.

To augment the model’s capacity for extracting feature information, this paper innovatively introduces the YOLO-BOS model, with its network architecture depicted in Figure 1. First, to enhance the capability of extracting salient foreground object features, we introduce an innovative BRSA attention mechanism strategically inserted prior to the SPPF module in the backbone network. By utilizing a bi-level routing approach and adjusting the weights of various positions, this mechanism reinforces useful features, enabling flexible computational allocation and content attention. This, in turn, enhances the ability to identify and capture salient features amid complex backgrounds. Additionally, we modify the C2F module in the head network using ODConv, which expands the model’s receptive field and perception range, facilitating the capture of more useful features, particularly for small-scale objects. Furthermore, during the training process, we introduce the Shape-IOU loss function, which takes into account the impact of the shape and size of bounding boxes on bounding box regression. This results in more accurate bounding box predictions for objects of various shapes, enhancing the regression performance and subsequently improving the overall detection accuracy.

### 3.2. BRSA Mechanism

**Figure 2:**
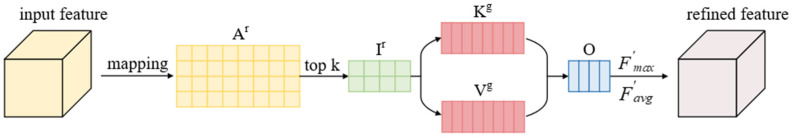
Structure diagram of the BRSA module.

In the backbone network, a novel BRSA attention mechanism is proposed, which is based on the BRA mechanism and capable of selectively filtering features according to task demands while leveraging SAM attention to adjust the significance of spatial positions for more accurate enhancement of useful features such as the shape, color, and texture of the target vehicle, therefore augmenting the model’s capacity to identify and capture salient features in complex backgrounds. Figure 2 illustrates the working process of BRSA: Initially, query (Q), key (K), value (V) and the adjacent matrix $A^{r}$ are obtained through linear mapping. Subsequently, a top-k routing attention mechanism is applied to $A^{r}$ to generate the routing index matrix $I^{r}$, which is then utilized to aggregate K and V into $K^{g}$ and $V^{g}$. Then, token-to-token attention is applied to Q, $K^{g}$, and $V^{g}$ to produce O. Finally, max pooling ($F_{max}^{\prime }$) and average pooling ($F_{avg}^{\prime }$) operations are performed to extract the final feature representation.

#### 3.2.1. Bi-Level Routing Attention

**Figure 3:**
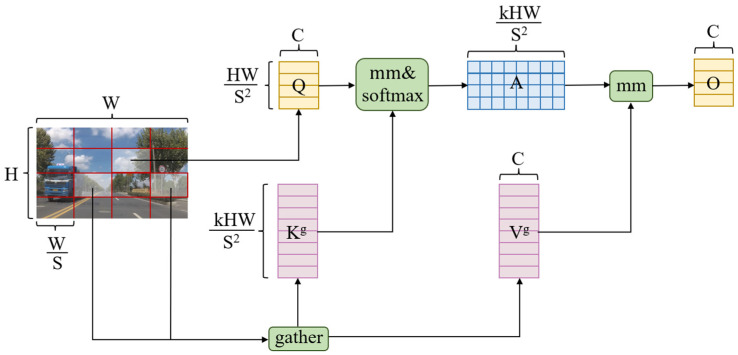
Structure diagram of the BRA module.

The Bi-level Routing Attention (BRA) mechanism represents a dynamic sparse attention mechanism capable of adjusting the distribution of attention dynamically based on input content, therefore conserving computational time and resources. Its core concept bifurcates the attention mechanism into two phases: the first phase filters out most irrelevant key-value pairs in coarse regions, retaining only a small portion of routing areas, therefore reducing the interference of redundant information. Subsequently, in the second phase, token-to-token attention is applied within the preserved routing areas to identify the most relevant regions. The operational process is depicted in Figure 3.

Initially, the input feature map is partitioned into regions, with the given two-dimensional feature map $X\in R^{H\times W\times C}$ divided into S×S non-overlapping regions. This division enables the transformation of X into $X^{r}\in R^{S^{2}\times \left(\frac{HW}{S^{2}}\right)\times C}$, therefore facilitating the linear mapping of $Q,K,V\in R^{S^{2}\times \left(\frac{HW}{S^{2}}\right)\times C}$, specifically:

(1) $$Q=X^{r}W^{q}$$

(2) $$K=X^{r}W^{k}$$

(3) $$V=X^{r}W^{v}$$

where $W^{q}$, $W^{k}$, $W^{v}$ represent the projection weights for Q,K,V, respectively.

To determine which areas each given subregion should focus on, average pooling is utilized to compute the regional features, yielding $Q^{r},K^{r}\in R^{S^{2}\times C}$. Subsequently, the region-to-region affinity adjacency matrix $A^{r}$ is obtained through the following formula:

(4) $$A^{r}=Q^{r}{\left(K^{r}\right)}^{T}$$

Each element within the adjacency matrix signifies the semantic relevance between two regions. Subsequently, by employing the top-k algorithm, the top-k connections in each region are preserved, effectively eliminating the portions with weaker associations:

(5) $$I^{r}=topIndex\left(A^{r}\right)$$

Here, $I^{r}$ denotes the routing index matrix, in which the $i^{th}$ row contains the K indices that are most strongly correlated with the $i^{th}$ region.

The subsequent phase involves the collection of keys and values for each query within the region and the computation of token-to-token attention. Specifically, for each query in the region, tensors for keys and values are collected as follows:

(6) $$K^{g}=gather\left(K,I^{r}\right)$$

(7) $$V^{g}=gather\left(V,I^{r}\right)$$

In Equations (6) and (7), the gather function extracts data from the input tensor based on the index matrix, generating a new, combined tensor. Attention is then focused on the collected information:

(8) $$O=Attention\left(Q,K^{g},V^{g}\right)+LCE\left(V\right)$$

Herein, the LCE function aims to parameterize V through deep convolution, with the kernel size set to 5.

Through Bi-level Routing Attention, the model can achieve more flexible computation allocation and content awareness, with selective focusing aiding in the capture of complex details and subtle features pertinent to vehicles, therefore enhancing the efficacy of vehicle object detection.

#### 3.2.2. Spatial Attention Mechanism

**Figure 4:**
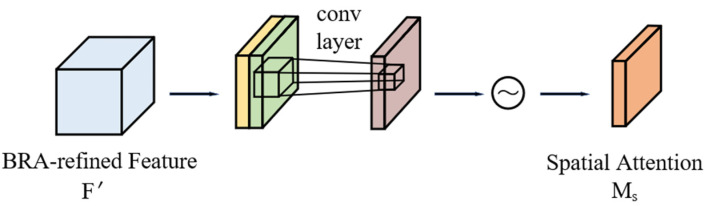
Structure diagram of the SAM module.

The Spatial Attention Module (SAM) focuses on the location information of the target, which can adjust the importance of spatial locations to more accurately enhance useful features. The operational process of the SAM is illustrated in Figure 4.

Initially, it generates two feature maps of H×W×1 dimension by applying max pooling and average pooling operations on the channel dimension to process the input feature maps obtained from the BRA module. These feature maps, concurrently capturing maximum and average values, empower the model with a more holistic comprehension of spatial distribution. Subsequently, these feature maps undergo concatenation, which is followed by a k×k convolution layer to extract intricate spatial features and formulate a spatial attention weight map. This map is then subjected to a sigmoid normalization function, ensuring its values lie within the range of [0, 1], thus yielding the output feature map of SAM. Ultimately, this refined output is element-wise multiplied with the original feature map, restoring its original H×W×C dimensionality while emphasizing salient spatial regions. The computational formulation underlying this SAM process is outlined as follows:

(9) $$M_{s}\left(F^{\prime }\right)=\sigma \left(f^{k\times k}\left(MaxPool\left(F^{\prime }\right);AvgPool\left(F^{\prime }\right)\right)\right)$$

where $f^{k\times k}$ denotes the convolution operation.

### 3.3. ODConv

In this work, we incorporate the ODConv after the C2F module in the head network. This particular convolution has the capability to adaptively adjust its parameters based on the input image, and it computes four distinct types of attention across all four dimensions of the kernel space—namely, the convolution kernel dimension, spatial dimension, input channel dimension, and output channel dimension—in a parallel fashion. By assigning different attention weights to each convolution kernel, this approach enhances the model’s capability to acquire input information and capture contextual cues effectively. Its definition is given by Equation (10) as follows:

(10) $$y=\left(\alpha_{\omega 1}⊙\alpha_{s1}⊙\alpha_{c1}⊙\alpha_{f1}⊙W_{1}+\ldots +\alpha_{\omega n}⊙\alpha_{sn}⊙\alpha_{cn}⊙\alpha_{fn}⊙W_{n}\right)*x$$

where $\alpha_{\omega i}\in R$ denotes the attention weight in the convolution kernel dimension, $\alpha_{si}\in R^{k\times k}$ represents the attention weight along the spatial dimension, $\alpha_{ci}\in R^{c_{in}}$ signifies the attention weight across the input channel dimension, and $\;\alpha_{fi}\in R^{c_{out}}$ indicates the attention weight along the output channel dimension. The attention weights in these four dimensions are computed by the multi-head attention module $\pi_{i}\left(x\right)$ The symbol ⊙ denotes the element-wise multiplication operation along different dimensions of the kernel space.

**Figure 5:**
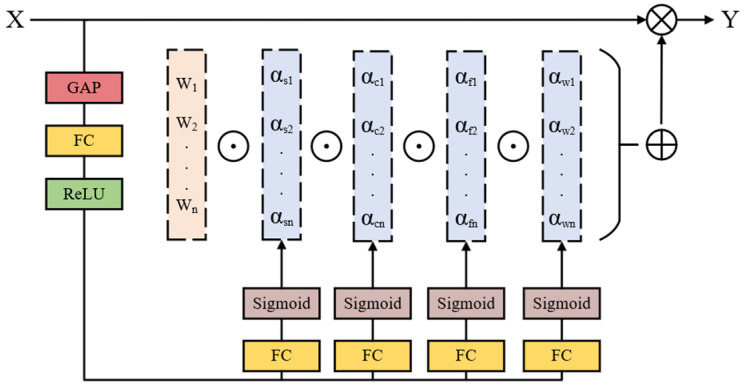
The schematic diagram of ODConv.

The schematic diagram of ODConv is presented in Figure 5. Initially, the input feature map X undergoes processing through a global average pooling (GAP) layer to diminish its feature dimensions. Subsequently, it is passed through a fully connected (FC) layer followed by a ReLU activation module. The processed features are then distributed into four parallel branches, each comprising a fully connected layer and a sigmoid function, to derive four distinct types of attention weights: $\alpha_{\omega i}$, $\alpha_{si}$, $\alpha_{ci}$, $\alpha_{fi}$. These attention weights are individually multiplied with the convolution kernels, enabling adaptive adjustments to the shape and weights of the kernels based on the content of the input features. Ultimately, the refined output feature map Y is obtained.

**Figure 6:**
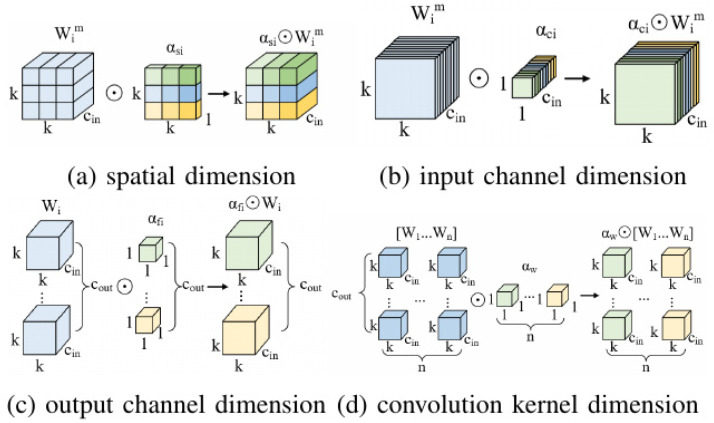
The four types of dimensional attention in the ODConv.

More specifically, Figure 6 depicts the process of multiplying the four types of dimensional attention in the kernel space by the convolution kernels. This procedure involves adjusting the weights of the convolution along the dimensions of the convolution kernel, spatial dimension, input channel dimension, and output channel dimension, respectively. These four attention mechanisms complement and cooperate, enabling the model to exert different dimensional influences on the input data across various dimensions. This enhances the convolutional neural network’s adaptability to variations in input data features and provides more flexible convolutional operations for processing complex and diverse data, therefore better accommodating the intricate and ever-changing conditions of road targets.

### 3.4. Shape-IOU

In urban traffic-dense scenarios, the shape and size of bounding boxes for road vehicles vary significantly due to factors such as occlusion and distance. The Shape-IOU loss function not only takes into account the geometric relationship between the predicted box and the ground truth box but also comprehensively considers the shape and scale factors of the bounding boxes [33]. Therefore, to achieve more accurate bounding box regression, we adopt the Shape-IOU loss function as the loss function for bounding box regression.

**Figure 7:**
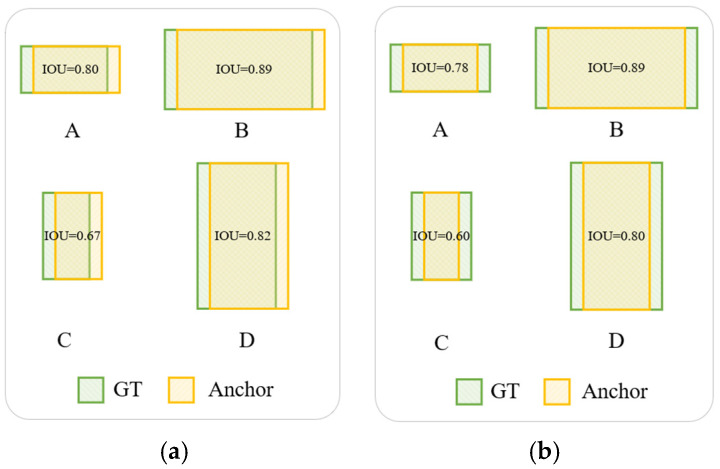
An illustration of regression affected by the shape and size of the bounding box themselves. (**a**) All bounding box regression samples possess the same deviation; (**b**) All bounding box regression samples possess the same shape deviation.

As illustrated in Figure 7, the shape and size of the bounding boxes themselves will affect the IOU value. Concretely, in Figure 7a, all bounding box regression samples exhibit the same deviation, with a shape deviation of 0. Conversely, in Figure 7b, all bounding box regression samples possess the same shape deviation, specifically with a deviation of 0. Within each set of figures, it is observed that samples A and B share the same shape, while samples C and D share another identical shape. Samples A and C are of the same size, and samples B and D are also of the same size, which is larger than that of A and C. It is noteworthy that for bounding box regression samples of equal size, despite having the same deviation or shape deviation, their IOU values can still vary. Furthermore, deviations or shape deviations along the shorter side of the bounding box exert a more significant impact on the IOU value. In the case of bounding box regression samples of the same shape, the IOU values of smaller-sized samples are more prone to being influenced by the shape of the ground truth (GT) box.

Therefore, taking into account the influence of the size and shape of the bounding box on its regression, the calculation method for Shape-IOU is defined as follows:

(11) $$IOU=\frac{\left|B\cap B^{gt}\right|}{\left|B\cup B^{gt}\right|}$$

(12) $$\omega \omega =\frac{2\times {\left(\omega^{gt}\right)}^{scale}}{{\left(\omega^{gt}\right)}^{scale}+{\left(h^{gt}\right)}^{scale}}$$

(13) $$hh=\frac{2\times {\left(h^{gt}\right)}^{scale}}{{\left(\omega^{gt}\right)}^{scale}+{\left(h^{gt}\right)}^{scale}}$$

(14) $$distance^{shape}=hh\times \frac{{\left(x_{c}-x_{c}^{gt}\right)}^{2}}{c^{2}}+\omega \omega \times \frac{{\left(y_{c}-y_{c}^{gt}\right)}^{2}}{c^{2}}$$

(15) $$\Omega^{shape}=\underset{t=\omega ,h}{\sum }{\left(1-e^{-\omega_{t}}\right)}^{4}$$

(16) $$\omega_{\omega }=hh\times \frac{\left|\omega -\omega^{gt}\right|}{\max\left(\omega ,\omega^{gt}\right)}$$

(17) $$\omega_{h}=\omega \omega \times \frac{\left|h-h^{gt}\right|}{\max\left(h,h^{gt}\right)}$$

where scale denotes the scaling factor related to the size of the targets in the dataset, ranging from 0 to 1.5, while ω, h, $\omega^{gt}$, and $h^{gt}$ signify the width and height of the predicted and the ground truth bounding boxes, respectively. ${\left(\omega^{gt}\right)}^{scale}$ and ${\left(h^{gt}\right)}^{scale}$ represent $\omega^{gt}$ and $h^{gt}$ adjusted by the scaling factor. $x_{c}$, $y_{c}$, $x_{c}^{gt}$, and $y_{c}^{gt}$, respectively, denote the center coordinates of the predicted bounding box and the ground truth bounding box. c stands for the length of the diagonal of the minimum bounding rectangle enclosing both the predicted bounding box and the ground truth bounding box. ωω and hh, respectively, indicate the weighting coefficients in the horizontal and vertical directions, with their values being related to the shape of the ground truth bounding box. The $distance^{shape}$ represents the distance loss function while $\Omega^{shape}$ denotes the shape loss function. The corresponding bounding box regression loss, denoted as $L_{Shape-IOU}$, is formulated as follows:

(18) $$L_{Shape-IOU}=1-IOU+distance^{shape}+0.5\times \Omega^{shape}$$

## 4. Experiments

### 4.1. Datasets

**Figure 8:**
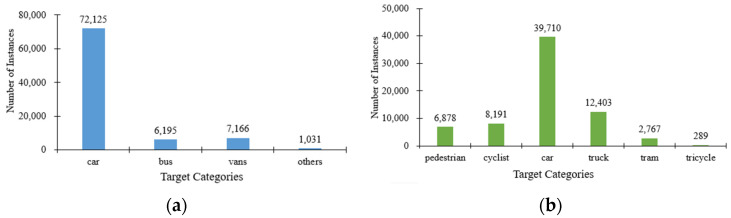
The distribution of instances for the datasets. (**a**) UA-DETRAC; (**b**) SODA10M.

The primary dataset selected for the experimental section of this paper is the UA-DETRAC dataset, which was collected from 24 road overpasses located in Beijing and Tianjin, China. This comprehensive dataset comprises over 10 h of video footage and encompasses more than 1.21 million object bounding boxes. The videos were captured with a frame rate of 25 fps and a resolution of 960 × 540 pixels. Each image in the dataset contains multiple vehicles of different types, exhibiting varying degrees of occlusion and truncation [34]. Furthermore, the dataset is annotated with four distinct label categories, namely car, bus, vans, and others. Considering that the dataset contains multiple continuous time-series videos and the differences in vehicles and background changes between adjacent video frames are not significant, directly utilizing it for object detection model training may result in data redundancy. Therefore, to enhance the model’s generalization ability and reduce training time, one frame is extracted from every ten frames. Subsequently, the processed dataset is divided into a training set and a validation set at a ratio of 8:2, yielding 8639 images for training and 2231 images for validation. The specific distribution of each category, as depicted in Figure 8a, reveals a clear dominance of the car category with a substantial count of 72,125 instances. This is followed by a gradual decline in the number of buses, vans, and other categories, exhibiting a pronounced long-tail effect.

In addition, to validate the extensive applicability of the proposed model, this study also performs supplementary verification on the SODA10M dataset. This dataset, jointly released by Huawei Noah’s Ark Lab and Sun Yat-sen University [35], encompasses road scene images from more than 30 cities across China. It consists of 20,000 labeled images with a resolution of 1920 × 1080 pixels, primarily classified into six categories: Pedestrian, Cyclist, Car, Truck, Tram, and Tricycle. In this experiment, 10,000 images are randomly selected from the dataset and divided into training, validation, and testing sets in a ratio of 8:1:1. Figure 8b illustrates the specific quantities of six label categories, where the number of cars is the highest, whereas the number of tricycles is the lowest. This distribution characteristic aligns with the popularity and inherent features of current transportation modes.

### 4.2. Experimental Settings

To ensure the validity of the experiments, all experiments presented in this work were conducted in a consistent operating environment, with specific configurations detailed in Table 1. In pursuit of achieving the best training outcomes, the models were trained for 100 epochs on the UA-DETRAC dataset and for 200 epochs on the SODA10M dataset. For both datasets, mosaic data augmentation was disabled during the final 10 epochs. The remaining training parameters were kept consistent across all experiments and are provided in Table 2 for reference.

### 4.3. Evaluation Metrics

In vehicle detection tasks, the primary metrics used to evaluate model performance include Precision (*P*), Recall (*R*), Mean Average Precision (*mAP*), and Frames Per Second (FPS). In this experiment, mAP@0.5 and mAP@0.5:0.95 serve as the main reference indices for performance, with their values positively correlated with the model’s effectiveness. Specifically, mAP@0.5 refers to the average of the Average Precision (*AP*) for each category when the IOU is set at 0.5, while mAP@0.5:0.95 denotes the average *AP* for each category when the IOU threshold ranges from 0.5 to 0.95 in increments of 0.05. The corresponding computational formulas are as follows:

(19) $$P=\frac{TP}{TP+FP}$$

(20) $$R=\frac{TP}{TP+FN}$$

(21) $$AP=\int_{0}^{1}PR\;dr$$

(22) $$mAP=\frac{1}{N}\overset{N}{\underset{i=1}{\sum }}AP_{i}$$

where *TP* denotes the number of correctly detected items, *FP* represents the number of vehicles incorrectly predicted, *FN* is the number of actual vehicles not detected, *AP* signifies the area enclosed by the *PR* curve, and *N* denotes the total number of categories in the dataset.

### 4.4. Impact of Different Modules on Model Performance

#### 4.4.1. Effect Verification of BRSA Mechanism

**Figure 9:**
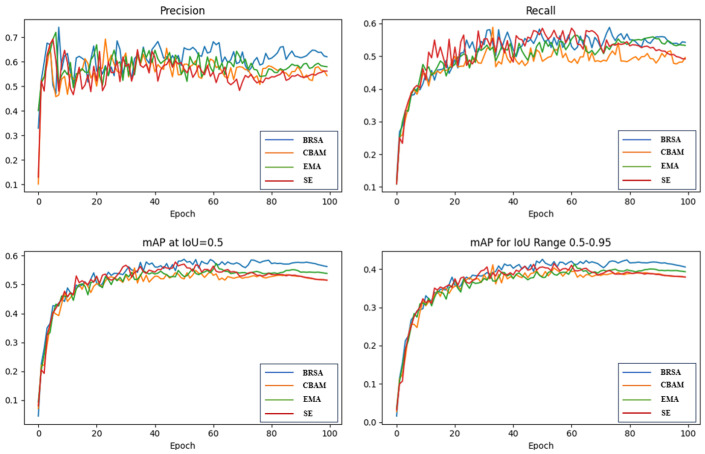
Experimental results of the comparison of different attention mechanisms.

To validate the effectiveness of the newly proposed BRSA attention mechanism, we integrated it alongside CBAM, EMA, and SE attention mechanisms at an identical location within the backbone network, precisely preceding the SPPF module. Comprehensive experiments were performed utilizing the UA-DETRAC dataset, and the resultant *P*, *R*, and *mAP* curves are depicted in Figure 9. The results unequivocally demonstrate that BRSA outperforms all other mechanisms across all evaluated curves.

Table 3 presents a detailed summary of the specific performance metrics observed after incorporating various attention mechanisms into the model. While the EMA and SE mechanisms exhibit faster detection speeds, they compromise on detection accuracy. Conversely, BRSA achieves an impressive mAP@0.5 of 58.8% and a noteworthy mAP@0.5:0.95 of 42.6%, outperforming all other evaluated attention mechanisms and demonstrating the most superior performance. This underscores the superior ability of our proposed attention mechanism to identify and capture salient features in complex backgrounds.

#### 4.4.2. The Impact of the Quantity and Placement of ODConv on Model Performance

In this research, we substituted two conventional convolution layers within the head network with ODConv modules, aiming to augment the convolutional neural network’s capacity to accommodate variations in the characteristics of input data, ultimately enhancing the precision of vehicle detection. Considering that the YOLOv8 network encompasses numerous convolution modules, we undertook a series of experiments to systematically analyze the influence of varying quantities and placements of ODConv modules on model performance.

Table 4 exhibits the influence of different quantities of ODConv modules in the backbone on model performance. It is evident that as the quantity of ODConv convolutions increases, the layer of the model escalates accordingly, accompanied by an improvement in the mAP@0.5 metric. However, when all convolutions in the backbone network are replaced with ODConv, the resulting model becomes excessively complex, leading to overfitting the training data and compromising its ability to generalize to new data, ultimately resulting in a decline in accuracy. As a result, we have chosen to replace only two standard convolutions in the head network with ODConv modules.

Table 5 delineates the effects of substituting standard convolutions with ODConv modules at various positions on model performance. In Group 1, a single convolution was replaced in both the head network and the backbone network. In Group 2, two convolutions within the backbone network were substituted. Group 3 encompassed the replacement of two convolutions in the head network. Group 4 signifies a combined experimental setup that integrates the model from Group 2 with the BRSA attention mechanism. Finally, Group 5 represents a combined experimental configuration that merges the model from Group 3 with the BRSA mechanism. From the experimental results, it can be observed that when replacing the convolutions in the backbone network, the model’s *P* improves, but the *R* decreases concurrently. Although both Group 2 and Group 3 exhibit comparably impressive performance in terms of mAP@0.5, Group 5, which combines the model from Group 3 with the BRSA attention mechanism, demonstrates superior performance. This could be attributed to the redundancy of the backbone network model in Group 4, which fails to provide additional valuable feature information. Consequently, we ultimately select the experimental configuration of Group 5, where two regular convolutions in the head network are replaced by the ODConv module.

#### 4.4.3. The Impact of Different Loss Functions on Model Performance

To evaluate the impact of different loss functions on model performance, ablation experiments were conducted using DIOU, EIOU, GIOU, SIOU, WIOU, Shape-IOU, and the baseline model. As shown in Table 6, the model’s GFLOPs remained unaffected by the substitution of loss functions. Notably, the EIOU loss function exhibited consistently poor performance across all metrics, potentially due to the need for refinement in the formulation of the Focal Loss term incorporated within EIOU. Additionally, while the SIOU loss function demonstrated an improvement in *P*, it suffered from a reduced *R*, thus compromising the overall detection effectiveness. Conversely, the model utilizing the Shape-IOU loss function achieved the highest performance on the dataset, with the highest mAP@0.5 and mAP@0.5:0.95 values, along with faster processing speeds. This robustly validates the effectiveness of incorporating the inherent attributes of bounding boxes in enhancing regression performance.

### 4.5. Ablation Studies

To validate the effectiveness of the various methods proposed in this paper, an ablation study was conducted, which comprised a total of 8 groups. To ensure fairness across all experimental groups, the input images, training hyperparameters, and other experimental configurations were kept consistent throughout the experimental process. The comprehensive results of this ablation study are presented in Table 7, which indicates that the baseline YOLOv8n model exhibits a rather modest detection performance, marked by an mAP@0.5 of 56.5%. By individually integrating the BRSA and ODConv, as well as modifying the loss function within the model, we observe distinct improvements in *P*, *R*, and *mAP*. Notably, although the inclusion of the BRSA module introduces an increase in computational complexity, it concurrently yields a substantial enhancement in mAP@0.5 by 2.3 percentage points. When these modules are paired, we see further improvements in mAP@0.5, with gains of 2.8, 2.6, and 3.8 percentage points, respectively, over the baseline YOLOv8n. Remarkably, when all three enhancements are combined, *P* surges by 5.3 percentage points, *R* increases modestly by 0.7 percentage points, and both mAP@0.5 and mAP@0.5:0.95 exhibit significant improvements of 4.7 and 4.4 percentage points, respectively. This underscores the profound enhancement in detection capabilities achieved by our model. In conclusion, the ablation study results conclusively validate the efficacy of each of the proposed modules.

### 4.6. Comparisons with Other Methods

Table 8 presents a comparison of evaluation metrics across various categories in the UA-DETRAC dataset before and after model improvement. As can be observed from the table, the improved model demonstrates enhancements of varying degrees across all categories, particularly with the *mAP* for the “other” category increasing from 29.6% to 44.3%, representing an improvement of over 40%.

To evaluate the performance of the improved model, we conducted a comparison with a range of YOLO models as well as the latest end-to-end object detection model, RT-DETR [36]. The performance of these models was assessed using various metrics, including mAP@0.5, mAP@0.5:0.95, GFLOPs, and Params. The experimental results are presented in Table 9. Under the metrics of mAP@0.5 and mAP@0.50:0.95, YOLO-BOS exhibited significantly higher performance compared to the other models, surpassing the second-best method, YOLOv5m, by 3.9 percentage points and 3.8 percentage points, respectively. Although the P metric of YOLO-BOS was slightly lower than that of the YOLOv5m, YOLOv7, and YOLOv7-CHS [37] models, the Params of these three models were substantially larger than that of YOLO-BOS. In terms of the FLOPs metric, YOLO-BOS only exceeded YOLOv5s by 2.7G, yet it achieved a 6.2 percentage point improvement in the mAP metric. Furthermore, compared to the RT-DETR model, our model demonstrates significant advantages in both accuracy and speed. The experimental results substantiate that enhancing the feature-extraction capability of the model can notably improve its detection accuracy.

To further demonstrate the generalization capability of our model, we conducted a series of comparative experiments on the SODA10M dataset. Table 10 presents a comparison of evaluation metrics across various categories in this dataset before and after model improvement. Similarly, for the “tricycle” category, which has the smallest proportion of samples, the mAP@0.5 improved by 16.1%. This indicates that our model possesses strong learning capabilities. As shown in Table 11, YOLO-BOS demonstrates significant improvements in both mAP@0.5 and mAP@0.5:0.95 metrics compared to YOLOv8n. Furthermore, it outperforms the YOLOv5s and YOLOv7 series in terms of both detection performance and model size.

**Figure 10:**
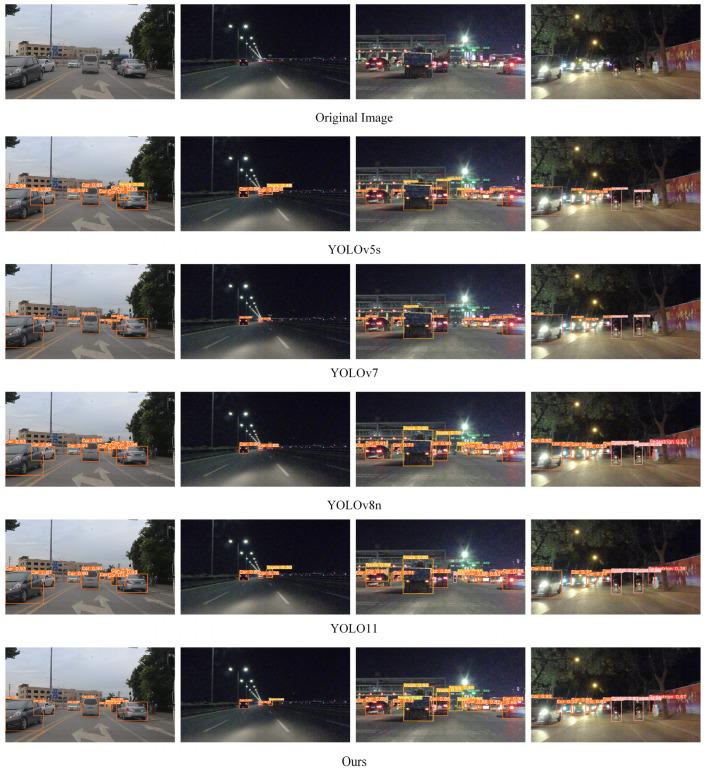
Detection results on the SODA10M dataset. The detection performance of different detection models on the SODA10M dataset.

For a more intuitive evaluation, we conducted a visual comparison of detection performance between our model and other detection models under varying lighting conditions and degrees of occlusion using scenes from the SODA10M dataset, as illustrated in Figure 10. The figure clearly indicates that YOLOv5s and YOLOv7 models suffer from elevated miss detection rates. Although YOLOv8n detects most targets, its performance deteriorates in complex scenarios. YOLO11 exhibits good performance across various scenarios, yet its detection efficacy in complex environments still lags behind that of YOLO-BOS, with instances of misdetection observed. This demonstrates that YOLO-BOS, with the three improvements proposed in this paper, significantly enhances feature-extraction capabilities and is less affected by factors such as lighting and occlusion, making it well-suited to various complex scenarios in urban road traffic.

## 5. Discussion

This paper proposes a novel YOLO-BOS vehicle-detection model to address the issues of missed detections, false detections, and reduced accuracy in vehicle detection caused by occlusion in dense scenarios. First, a novel BRSA attention mechanism is proposed, which can selectively filter features according to task requirements and utilize the SAM attention to adjust the importance of spatial positions, therefore more accurately enhancing useful features and improving the quality of feature extraction from input images. Second, the ODConv is integrated into the head network, which dynamically adjusts the size and weights of the convolution kernel across four dimensions of the kernel space, enabling better adaptation to the complex and varying conditions of road targets. Additionally, Shape-IOU is employed as the loss function instead of CIOU, which not only considers the geometric relationship between the predicted box and the ground truth box but also comprehensively takes into account the shape and scale factors of the bounding boxes, therefore enhancing the accuracy and robustness of vehicle target detection across different sizes. The feasibility of each improvement is demonstrated through ablation experiments, and comparative experiments are conducted on two different datasets to prove the generalization capability of the model. Although the proposed model has achieved notable improvements in detection accuracy, there is still considerable room for optimization in terms of computational complexity and inference speed. In our future work, we will focus on deeply optimizing the computational complexity and inference speed of the model while maintaining its detection accuracy.

## Figures and Tables

**Table 1:** Configuration settings for the experimental environment.

Type	Configuration
CPU	E5–2678 v3@2.50 GHz
GPU	NVIDIA GeForce RTX 2080 Ti
Operating system	Windows 10
RAM	128 GB
CUDA	11.6
Programming language	Python 3.8
Deep learning architecture	Pytorch 1.13.1

**Table 2:** Configuration of parameters in the experimental setup.

Parameters	Value
Image size	640 × 640
Batch size	32
Optimizer	SGD
Initial learning rate	0.01
Momentum	0.937
Weight decay	$5\times 10^{-4}$
Workers	8

**Table 3:** Experimental results on different attention mechanisms.

Module	mAP@0.5 (%)	mAP@0.5:0.95 (%)	Layers	FPS
CBAM	55.8	41.1	180	217.39
EMA	57.3	41.3	176	285.71
SE	56.9	41.5	175	285.71
BRSA	58.8	42.6	183	263.16

**Table 4:** The impact of the quantity of ODConv on model performance.

Quantity	P (%)	R (%)	mAP@0.5 (%)	mAP@0.5:0.95 (%)	Layers
0	59.2	57.7	56.5	40.6	168
1	61.8	55.3	56.8	40.4	183
2	57.4	57.4	57.6	41.6	193
3	60.3	52.8	54.2	39.9	204

**Table 5:** The impact of the placement of ODConv on model performance.

Group	P (%)	R (%)	mAP@0.5 (%)	mAP@0.5:0.95 (%)	Layers
1	64.2	53.6	56.0	41.0	194
2	64.9	57.1	57.5	41.8	194
3	57.4	57.4	57.6	41.6	193
4	63.5	57.1	58.6	43.0	209
5	66.8	54.3	59.3	43.5	208

**Table 6:** The impact of different loss functions on model performance.

Loss Function	FLOPs (G)	P (%)	R (%)	mAP@0.5 (%)	mAP@0.5:0.95 (%)
Baseline	8.1	59.2	57.7	56.5	40.6
DIOU	8.1	60.9	55.6	56.0	40.6
EIOU	8.1	55.3	54.1	54.8	39.5
GIOU	8.1	65.5	51.9	56.7	40.6
SIOU	8.1	67.1	51.7	57.0	40.6
WIOU	8.1	55.6	59.3	56.0	40.2
Shape-IOU	8.1	61.6	56.2	57.6	40.8

**Table 7:** Ablation experiments. In columns 2 through 4, the “√” sign indicates that the module in line 1 was added to the YOLOv8 network.

Group	BRSA	ODConv	Shape-IOU	P (%)	R (%)	mAP@0.5 (%)	mAP@0.5:0.95 (%)	FLOPs (G)	FPS
1				59.2	57.7	56.5	40.6	8.1	312.50
2	√			65.6	55.8	58.8	42.6	18.7	263.16
3		√		57.4	57.4	57.6	41.6	8.1	277.78
4			√	61.6	56.2	57.6	40.8	8.1	303.03
5	√	√		66.8	54.3	59.3 (+2.8)	43.5	18.5	285.71
6	√		√	60.6	61.1	60.3 (+3.8)	43.7	18.7	285.71
7		√	√	59.3	40.9	59.1 (+2.6)	40.9	8.1	277.78
8	√	√	√	64.5	58.4	61.2 (+4.7)	45.0	18.5	263.16

**Table 8:** Model detection results for different categories in the UA-DETRAC dataset.

Category	YOLOv8n				Ours			
	P (%)	R (%)	mAP@0.5 (%)	mAP@0.5:0.95 (%)	P (%)	R (%)	mAP@0.5 (%)	mAP@0.5:0.95 (%)
car	70.5	70.7	74.1	53.2	68.8	71.7	74.3	53.0
bus	71.1	72.0	72.3	52.8	74.5	72.9	74.9	55.8
vans	53.2	52.4	50.1	38.3	51.9	52.9	51.3	39.1
others	41.9	35.6	29.6	18.1	62.8	35.8	44.3	32.1
all	59.2	57.7	56.5	40.6	64.5	58.4	61.2	45.0

**Table 9:** Comparisons of different object detectors on the UA-DETRAC dataset.

Model	P (%)	R (%)	mAP@0.5 (%)	mAP@0.5:0.95 (%)	Params (M)	FLOPs (G)
YOLOv5s	59.3	55.5	55.0	39.2	7.02	15.8
YOLOv5m	70.6	53.3	57.3	41.2	20.87	47.9
YOLOv7	82.0	51.9	53.4	41.7	36.50	103.2
YOLOv7-CHS	77.1	448.1	52.6	36.0	33.56	40.3
YOLOv8n	59.2	57.7	56.5	40.6	3.00	8.1
YOLO11	61.8	51.7	55.9	40.2	2.58	6.3
RT-DETR	51.5	49.9	45.9	32.7	32.8	108.0
Ours	64.5	58.4	61.2	45.0	3.27	18.5

**Table 10:** Model detection results for different categories in the SODA10M dataset.

Category	YOLOv8n				Ours			
	P (%)	R (%)	mAP@0.5 (%)	mAP@0.5:0.95 (%)	P (%)	R (%)	mAP@0.5 (%)	mAP@0.5:0.95 (%)
pedestrian	69.2	28.8	39.5	18.5	57.0	32.7	39.1	17.7
cyclist	73.0	53.6	63.7	36.8	69.6	55.9	61.9	34.8
car	85.1	76.8	84.3	61.8	78.3	78.4	83.0	59.3
truck	77.7	66.3	74.1	52.1	76.3	70.1	76.0	51.8
tram	71.6	63.9	69.5	52.1	72.0	64.9	70.4	52.8
tricycle	60.2	31.2	34.3	25.1	69.4	40.1	50.4	30.4
all	72.8	53.4	60.9	40.1	70.4	57.0	63.5	42.1

**Table 11:** Comparisons of different object detectors on the SODA10M dataset.

Model	P (%)	R (%)	mAP@0.5 (%)	mAP@0.5:0.95 (%)	Params (M)	FLOPs (G)
YOLOv5s	79.3	48.6	56.6	35.7	7.03	15.8
YOLOv7	78.7	48.1	54.8	35.3	36.5	103.2
YOLOv7-tiny	65.9	35.3	40.3	25.8	6.02	13.1
YOLOv8n	70.8	53.4	60.9	40.1	3.0	8.1
YOLO11	75.6	53.8	62.0	42.2	2.58	6.3
RT-DETR	73.3	50.5	55.3	35.6	32.0	108.0
Ours	70.4	57.0	63.5	42.1	3.27	18.5

## Data Availability

The original contributions presented in the study are included in the article. Further inquiries can be directed to the corresponding author.
